# General practitioners’ views on retaining Singapore’s primary care doctors: a cross-sectional survey and qualitative analysis

**DOI:** 10.1186/s12875-022-01774-z

**Published:** 2022-07-01

**Authors:** Yang Fang, Michael Soljak, Shawn Lien Ler Tan, Stephen Peckham, Tze Lee Tan, Helen E. Smith

**Affiliations:** 1 Lee Kong Chian School of Medicine, Nanyang Technological University Singapore, 11 Mandalay Road, Singapore, 308232 Singapore; 2 Faculty of Medicine, Imperial College London, South Kensington Campus, London, SW7 2AZ UK; 3 Ministry of Health Holdings, 1 Maritime Square, Singapore, 099253 Singapore; 4 Centre for Health Services Studies, University of Kent, Canterbury, CT2 7NF UK; 5 The Edinburgh Clinic, Blk 306, #01-685, Choa Chu Kang Ave 4, Singapore, 680306 Singapore

**Keywords:** Primary care, General practice, General practioners, Family medicine, Healthcare workforce, Managed care

## Abstract

**Background:**

To support its ageing population and the increasing need for chronic care in the community, Singapore needs to boost the number of doctors in its primary care workforce. To better understand how to improve doctor retention and build a more robust primary care system, we conducted a cross-sectional survey with doctors in general practice and family medicine to explore their career satisfaction, their career plans, factors related to their plans to leave, and their view on retaining GPs in primary care.

**Methods:**

An anonymous online survey was distributed to general practitioners working in the public and private sectors. The survey contained questions on career satisfaction, career plans in the next 5 years, and factors important for retaining doctors in primary care. In addition, there were open-ended questions for respondents to elaborate on retention initiatives and other factors that may improve engagement among primary care doctors. Quantitative data was analyzed with descriptive statistics, principal component analysis, χ^2^ tests, t-tests, and Pearson’s correlations; qualitative data was analyzed thematically.

**Results:**

The survey was attempted by 355 general practitioners and completed in full by 303. The respondents were most satisfied with rapport with patients and their current professional role; they were least satisfied with the amount of paperwork and the status of general practice in society*.* In terms of their career plans in the next 5 years, 49/341 (14.4%) of the respondents plan to leave general practice permanently, 43/341 (12.6%) plan to take a career break, and 175/341 (51.3%) plan to reduce their clinical hours. Higher remuneration, recognizing general practice and family medicine as a medical specialty, and reducing the litigious pressures on medical practice were rated as the most important factors for retaining primary care. Free-text responses also revealed a growing dissatisfaction with the Third-Party Administrators that manage insurance arrangements.

**Conclusion:**

While the proportion of doctors who intend to leave is smaller than that reported in overseas studies, our findings highlight an urgent need for targeted interventions to engage and retain primary care doctors. Increasing recognition and support for general practitioners and their professional practice may contribute to strengthening community care for the ageing population.

## Background

Strong primary care contributes to more efficient care and better outcomes [1]; its role in health care becomes even more crucial as the population ages and the need for chronic care increases. To care for its rapidly ageing population, Singapore has recognized the need to strengthen its primary care workforce [2], and this is evidenced in the Health Care Manpower Plan 2020 [3]. One of the core strategies of the Plan is to train more doctors to work in primary care i.e. general practice and family medicine (GPFM). This is laudable but given the growing challenges faced by doctors caring for a populace with increasingly complex medical needs, attention must also be given to the retention and recruitment of doctors within the primary care workforce. It is important to understand their current perceptions of their profession and what can be done to engage and retain them in practice.

A recent systematic review of 25 studies showed that countries throughout the world have high turnover intentions among general practitioners (GPs) [4]. In the United Kingdom, where the government target for GP recruitment has not been met, the intention to quit is driven by low morale [5], work volume and intensity [6], concerns about risks and future of general practice [7]. In a national survey in China, over 70% of GPs expressed moderate to strong turnover intentions, which were associated with low job satisfaction and burnout [8]. In Singapore, GPs have also voiced dissatisfaction about keen market competition with easy access for patients to specialists [9], lower status and pay compared to specialists [10], and the difficulties in providing “managed care” [11], which requires dealing with multiple third-party administrators [12]. Burnout amongst private GPs in Singapore, who tend to work alone and need to ensure the clinical and financial viability of their practices without external resources, have also been raised as an issue of concern [13].

Primary care in Singapore is provided in two settings; by 20 government polyclinics and approximately 1800 private practices (solo practices or group practices of varying sizes). Private practices provide approximately 80% of all primary care services, but polyclinics provide a disproportionate amount of care for chronic disorders [14]. GPs in polyclinics work in teams consisting of nurses and allied health professionals, have greater opportunities to pursue more varied portfolios in teaching and research, and have little control over their patient load. In contrast, private GPs tend to work alone with little support from other health professionals; they have more control over their patient load but find it more challenging to engage in teaching and research. Prior work has categorized Singapore as a “low” primary care country, indicated by lower earnings of primary care physicians compared to specialists, lack of 24-h primary care services, and the standard of family medicine in academic departments [10]. This finding does not bode well for a country in which 25% of its population is expected to be aged 65 or above by 2030. Therefore, we surveyed Singaporean GPs on their career satisfaction, their career plans, factors related to their plans to leave, and their views on retaining GPs in primary care. Our findings will help with planning a more robust primary care system and improve GP retention.

## Methods

### Survey design

This survey was part of a larger mixed-methods study examining the recruitment, re-engagement, and retention of GPs in Singapore. Qualitative interviews with 40 GPs (20 practicing (16 in the private and 4 in the public sector), 20 no longer practicing) [15] were conducted to generate topics for this survey. The questionnaire content was also informed by the existing literature, and questions used in previous GP workforce surveys elsewhere [5, 6, 16]. The 19-item questionnaire was formatted with multiple-choice, check-box, as well as open-ended questions for free-text responses. Refinements were made after the questionnaire was reviewed for clarity, content, appropriateness by 10 practicing GPs on the study advisory panel, and format and by two academic health psychologists for content validity [17].

The questionnaire focused on three areas of the participants’ careers: i) satisfaction with different aspects of their careers (0 = least satisfied; 10 = most satisfied), ii) career plans for the next 5 years (including the intention to and reasons for leave practice or to take a career break), and iii) importance of different factors for retaining doctors in GPFM (0 = least important; 10 = most important). Respondents were also able to share their thoughts on other retention initiatives and other comments in the open-ended questions. The characteristics of respondents’ practice, professional experience, and demographics were collected.

### Survey distribution

The survey was administered on the online survey platform Qualtrics. To distribute the survey widely to GPs practicing in Singapore, we contacted the three healthcare groups that manage the twenty polyclinics in Singapore (all of whom agreed to help with distribution), and ten Primary Care Networks that connects over 600 private GP clinics in Singapore (five PCNs responded with varying offers of distribution help), and public agencies liaising with private primary care clinics. Since telephone contact of private GP clinics are publicly available but not their email addresses, phone calls were made to the clinics and 433 valid email addresses were obtained for distributing a survey invitation. A second invitation was sent after 2 months. GPs on the study’s advisory panel also helped to distribute the survey through their personal networks. Informed consent was obtained online and no personally identifiable information was collected.

### Data analyses

Descriptive statistics were used to present an overview of the quantitative data and to identify the items rated as most important by the participants. Where applicable, principal component analysis with Promax rotation [18] was used to reduce questions with multiple items into fewer dimensions to increase explanatory power. Chi-square tests of association were performed to examine association between two categorical variables. T-tests were performed to examine differences by age and across sectors (public vs private). Stepwise logistic regression was conducted to identify the factors that predict the participants’ likelihood of leaving GPFM in the next 5 years. All statistical analyses were conducted in SPSS Version 22 [19].

Free-text data collected in response to open-ended questions underwent thematic content analysis [20]. The first coder read through all of the responses and identified the preliminary codes and themes in a subset of the responses. Then, a second coder checked the coding of the subset and resolved any difference with the first coder. All responses were coded with the revised coding scheme and themes were linked to the quantitative analyses to supplement the interpretation of the data.

Ethics approval was obtained from the Institutional Review Board, Nanyang Technological University (IRB-2018–07-007).

## Results

Three hundred and fifty-five doctors responded to the questionnaire (indicated by their completion of the first question). Given that there are about 80% primary care services in Singapore are provided by 1800 private general practices in Singapore primarily staffed by a single GP, we estimate total population of practicing GPs to be approximately 2250. This results in a response rate of 16%, with 85% (*N* = 303) completing the survey in full. The majority of the responses were collected between June and December 2020. The characteristics of the sample are shown in Table 1. Approximately 16% of the respondents work in the public primary health sector, slightly lower than the 20% estimate officially reported.

**Table 1:** Characteristics of GP participants

	**N**	**Percent**
Age group		
≤ 30	12	4.0%
31 to 35	32	10.6%
36 to 40	44	14.5%
41 to 45	36	11.9%
46 to 50	48	15.8%
51 to 55	52	17.2%
56 to 60	36	11.9%
61 to 65	25	8.3%
> 65	18	5.9%
Gender		
Male	205	65.7%
Female	99	31.7%
Prefer not to say	8	2.6%
Ethnic group		
Chinese	273	87.5%
Malay	5	1.6%
Indian	25	8.0%
Others	9	2.9%
Practice type (multiple selection)		
Polyclinic (Public)	45	
Private Group	135	
Private Solo	149	
Community Hospital (Public)	5	
Freelance/Locum	20	
Others	20	
Roles (multiple selection)		
Clinical practice	350	
Teaching	72	
Mentoring	56	
Research	25	
Administration	143	
Leadership	79	
Others	7	
Highest qualification (related to FM)		
MBBS/MD	156	43.9%
Graduate Diploma in Family Medicine*	107	30.1%
Master of Medicine in Family Medicine	53	14.9%
Fellowship in Family Medicine	37	10.4%
Others	2	.6%
*A two-year training program in family medicine for practicing doctors		
Years of experience		
< = 5 years	45	12.7%
6 to 10 years	55	15.5%
11 to 15 years	41	11.5%
16 to 20 years	57	16.1%
21 to 25 years	56	15.8%
26 to 30 years	52	14.6%
31 to 35 years	22	6.2%
36 to 40 years	10	2.8%
41 to 45 years	8	2.3%
> 45 years	9	2.5%
Hours worked per week		
< 10	3	.8%
10–19	22	6.2%
20–29	39	11.0%
30–39	71	20.0%
40–49	145	40.8%
≥ 50	75	21.1%

### Career satisfaction

When asked about satisfaction with different aspects of their career on a scale of 0 (least satisfied) to 10 (most satisfied), the participants were most satisfied with *rapport with patients* and their *current professional role*, both receiving a mean score above 7. Conversely, the participants were least satisfied with *paperwork* and the *status of GPFM in society*, both receiving a mean score below 5. The mean and standard deviation (SD) of each item are shown in Table 2. There were no gender differences on any of the items.

**Table 2:** Satisfaction with different aspects of the GPFM career

			Components		
	Mean	Standard Deviation (SD)	GP Role	Clinical work and patient care	Relationships and status
Physical resources (such as premises and equipment)	5.73	2.31	0.902		
Working hours	5.44	2.40	0.874		
Staffing level	5.55	2.21	0.797		
Status of GP/FM in society	4.82	2.42	0.781		
Intensity of workload	5.45	2.25	0.752		
Paperwork	4.36	2.58	0.681		
Income	5.80	2.43	0.663		
Learning opportunities	6.40	2.14	0.432		
**Rapport with patients**	**7.31**	**1.74**		0.971	
Time for patients	6.27	2.13		0.900	
Recognition from patients	6.37	2.36		0.658	
**Autonomy**	**6.92**	**2.38**		0.611	
**Current professional role**	**7.07**	**1.87**		0.539	
Case mix (range of patient problems)	6.53	2.07		0.53	
Intellectual stimulation	6.37	2.19		0.424	
Relationship with management/ superiors	6.40	2.70			0.997
**Relationship with colleagues**	**6.88**	**2.11**			0.834
Recognition from superiors	5.45	2.87			0.760
Career progression opportunities	5.12	2.93			0.698
Recognition from colleagues	5.82	2.55			0.654
Distribution of workload among colleagues	5.66	2.48			0.560

For Mean and SD, 0 = least satisfied, 10 = most satisfied. The most highly rated items are denoted in bold

A principal component analysis (Promax rotation) to explore the factors underlying the list of items revealed a three-component solution that explained 65.6% of the variance (Table 2). Sampling adequacy was confirmed by a Kaiser–Meyer–Olkin (KMO) measure of 0.935 and a significant (*p* < 0.05) Bartlett test of sphericity. The “GP role” component relates to job and career characteristics. The items include *physical resources (e.g., premises and equipment), working hours, staffing level, status of GPFM in society, intensity of workload, paperwork, income, and learning opportunities*. The “clinical work and patient care” component relates to the clinical aspects of the job and interaction with patients. The items include *rapport with patients, time for patients, recognition from patients, autonomy, current professional role, case mix, and intellectual stimulation.* The “relationships and status” component relates to aspects of their job that involves interaction and coordination with colleagues and superiors. The items include *relationship with management/superiors, relationship with colleagues, recognition from superiors, career progression opportunities, recognition from colleagues,* and *distribution of workload among colleagues*.

### Career plan in next five years

In terms of their career plans in the next 5 years, 49 GPs (14.4%) indicated a plan to leave GPFM permanently, over half of them (26 (53.0%)) intended to retire. In addition, 175 (51.3%) indicated an intention to reduce their hours of clinical work, and 43 (12.6%) intend to take a career break (Table 3). Given the differences in the nature of work and career paths of doctors in public and private settings, we examined whether career plans of doctors may differ across the settings. To correct for multiple comparison, we computed the adjusted α-level (i.e., *p*-value) for rejecting the null hypothesis using Holm’s procedure [21]. We found that those working in public settings were more likely to increase teaching (*p* < 0.001), training (*p* = 0.001), or research (*p* = 0.001) responsibilities. In contrast, participants working in private settings were more likely to reduce clinical work hours (*p* = 0.001). This might be due to participants in the private settings being significantly older than participants in public settings (*p* < 0.001) in our sample, which is consistent with the actual population of GPs in Singapore. The other career plans showed no significant association with public versus private work setting.

**Table 3:** Career plans of Singapore GPs in the next 5 years

	All (341) N (%)	Public (52) N (%)	Private (289)	Differences across public and private (χ^2^ and *p*-value)	Test of significance against adjusted α (Holm’s procedure)
Leave GP/FM permanently	49 (14.4%)	2 (3.8%)	47 (16.3%)	χ2 = 5.52 (*p* = .019)	NS
Take a career break	43 (12.6%)	6 (11.5%)	37 (12.8%)	NS	NS
Work abroad	21 (6.2%)	4 (7.7%)	17 (5.9%)	NS	NS
Pursue further professional studies	65 (19.1%)	16 (30.7%)	49 (17.0%)	χ2 = 5.45 (*p* = .020)	NS
Participate in training programmes	83 (24.3%)	19 (36.52%)	64 (22.1%)	χ2 = 4.96 (*p* = .026)	NS
Reduce hours of clinical work	175 (51.3%)	14 (26.9%)	161 (55.7%)	χ2 = 14.618 (*p* < .001)	**Sig (adj α = .002)**
Increase hours of clinical work	16 (4.7%)	6 (11.5%)	10 (3.5%)	χ^2^ = 6.43 (*p* = .011) ^#^	NS
Reduce management responsibilities	76 (22.3%)	7 (13.5%)	69 (24.1%)	NS	NS
Increase management responsibilities	43 (12.6%)	12 (23.1%)	31 (10.7%)	χ2 = 6.10 (*p* = .014)	NS
Reduce teaching responsibilities	8 (2.3%)	0 (0.0%)	8 (2.8%)	NS	NS
Increase teaching responsibilities	46 (13.5%)	20 (38.4%)	26 (9.0%)	χ2 = 32.79 (*p* < .001)	**Sig (adj α = .002)**
Reduce training responsibilities	8 (2.3%)	1 (1.9%)	7 (2.4%)	NS	NS
Increase training responsibilities	28 (8.2%)	12 (23.1%)	16 (5.6%)	χ2 = 11.20 (*p* = .001)	**Sig (adj α = .002)**
Reduce research responsibilities	6 (1.8%)	3 (5.8%)	3 (1.0%)	χ2 = 5.71 (p = .017) ^#^	NS
Increase research responsibilities	23 (6.7%)	9 (17.3%)	14 (4.8%)	χ2 = 11.87 (*p* = .001) ^#^	**Sig (adj α = .002)**
Others (status quo, business ventures, etc.):	55	5	50		

Multiple selections are allowed for this question. NS denotes not significant

^#^At least one cell contains minimum expected cell count of less than five, hence rendering the Chi-sq Test unsuitable. Therefore, the significance of the Fisher’s exact test is reported instead

Participants planning to leave GPFM within the next 5 years were asked to rate the importance of 17 items (Table 4). The top four reasons were *time spent on unimportant tasks, reduced job satisfaction, long working hours,* and *lack of professional support*.

**Table 4:** Importance of different factors to GPs’ plans to leave

	Mean	SD
Time spent on unimportant tasks	6.89	2.92
Reduced job satisfaction	6.64	3.02
Long working hours	5.90	3.06
Lack of professional support	5.90	2.78
Lack of time for personal growth eg. training/ further education/ research	5.76	3.29
Intensity of workload	5.69	2.81
Volume of workload	5.51	3.19
Lack of career progression opportunities	5.21	3.08
Complexity of patient problems	4.02	2.74
Lack of time for patient contact	3.90	3.04
Lack of continuity of care for patients	3.85	2.71
Lack of innovation opportunities	3.70	3.37
Lack of research opportunities	3.56	3.33
Lack of teaching opportunities	3.47	3.09
Lack of mentoring opportunities	3.11	2.89
Poor working relationship with colleagues	2.74	2.29

For Mean and SD, 0 = least important, 10 = most important

Similarly, those participants who indicated a plan to take a career break within the next 5 years were asked to indicate their reasons for doing so (Table 5). The two most frequently selected reasons were *travel* and *financial security*. The most frequently selected ideal break length was less than a year (46%). Contrary to the common perception that females are more likely to leave to workforce to look after children or dependents, no significant gender differences were observed in our sample.

**Table 5:** Reasons for planning to take a career break in the next 5 years

Reason	N
Travel	18
Financially secure	10
Others (rest, tired, relieve stress):	9
Looking after young children	7
Looking after dependent/s	7
Work abroad	7
Health issues	7
Study abroad	3
Starting a family	2
Expanding my family	1

### Promoting the retention doctors within GPFM

When rating the importance of different factors needed to improve retention of doctors within GPFM on a scale of 0 (least important) to 10 (most important), the most highly rated factors were *greater commitment to protect professional practice, less litigious environment for medical practice, higher remuneration,* and *recognizing GPFM as a specialty* (Table 6).

**Table 6:** Importance of different factors in retaining doctors within GPFM

	All		Public		Private		Difference between private and public (t- and *p*- values)	Test of significance against adjusted α (Holm’s procedure)
	Mean	SD	Mean	SD	Mean	SD		
Greater commitment by relevant bodies to protect professional practice	8.63	1.86	8.29	1.62	8.69	1.89	NS	NS
Less litigatious environment for doctors to practise medicine	8.62	1.95	8.21	1.95	8.69	1.93	NS	NS
Higher remuneration	8.22	1.91	8.35	1.70	8.20	1.94	NS	NS
Recognise General Practice/Family Medicine as a specialty	8.05	2.47	8.00	2.30	8.05	2.50	NS	NS
More training for GPs and family physicians to handle certain illnesses within primary care setting	7.67	2.23	7.58	2.16	7.68	2.25	NS	NS
More professional support	7.65	1.89	7.57	1.90	7.66	1.89	NS	NS
Greater continuity of care for patients	7.53	2.01	7.65	1.74	7.50	2.05	NS	NS
Reduced administrative tasks	7.52	2.35	7.57	2.40	7.24	2.04	NS	NS
Improved working culture that enhances collegiality	7.49	1.93	7.46	1.64	7.50	1.98	NS	NS
Greater job autonomy	7.34	2.11	7.58	1.90	7.29	2.15	NS	NS
More recognition of clinical and other contributions	7.33	2.11	7.18	1.81	7.36	2.16	NS	NS
Additional annual leave	7.29	2.44	7.75	1.97	7.20	2.51	NS	NS
More career progression opportunities	7.12	2.29	7.61	1.93	7.04	2.34	NS	NS
Longer consultation time with patients	7.1	2.29	8.10	2.00	6.92	2.30	t = 3.38 (*p* = .001)	**Sig (adj α = .002)**
Protected time for education/ training/ research	6.82	2.36	7.79	2.07	6.65	2.37	t = 3.14 (*p* = .002)	**Sig (adj α = .002)**
Reduced intensity of workload	6.42	2.33	7.59	1.89	6.21	2.35	t = 3.97 (*p* < .001)	**Sig (adj α = .002)**
Reduced volume of workload	6.23	2.43	7.55	1.94	5.99	2.44	t = 4.31 (*p* < .001)	**Sig (adj α = .002)**

For Mean and SD, 0 = least important, 10 = most important

Thematic analyses of the free-text responses also stressed the importance of higher remuneration and more recognition to retain doctors within GPFM, for example, *“greater recognition by MOH as a specialty in itself. Higher pay.”* (P64, male, public); *“Higher /similar salary compared to other specialist”* (P45, female, public). Besides recognizing GPFM as a specialty by official bodies, GPs also emphasized a need for greater recognition of GPFM by the public and other doctors: “*that the public recognize that we are equipped with the knowledge and skills to handle many diseases at the primary care level that do not need to be referred to specialists”* (P324, female, private); *“More public education from MOH regarding the expertise of a trained Family Physician, and why public should not look to seeing a specialist first”* (P158, male, public).

### Comparison of retention initiatives needed in the public and private sectors

Given the differences in the nature of work in public and private sector, understanding how retention factors may vary for doctor in the two different settings is important. Comparing changes needed to enhance retention between private and public sectors, GPs in public settings gave higher importance ratings to *reduction of volume (t* = 4.31*, p* < 0.001*)* and *intensity of workload (t* = 3.97*, p* < 0.001*), longer consultation times (t* = 3.38*, p* = 0.001*)* and *protected time for education/training/research (t* = 3.14*, p* = 0.002*)* than those in private settings. The other retention policies showed no differences across sectors (Table 6).

### Dissatisfaction with private Third-Party Administrators

Private third-party administrators (TPAs) were not included in the questionnaire as a possible reason for career change. However, the negative impact of TPAs were spontaneously reported in 53 free text comments. TPAs facilitate managed care (referring broadly to methods that reduces cost of providing healthcare). Employers often outsource the management of employee healthcare benefits to TPAs, who then enter into contracts with healthcare providers to provide healthcare services within a fee structure. Study participants described three major ways in which TPAs affect their practice negatively: financial exploitation, professional belittlement, and reducing the quality of patient care they were able to provide.

The reimbursements allowed by some TPAs were perceived to be too low to meet the cost of consultations, with negative impact on the practice’s income and profit margin.

*“TPA and insurance companies make GP lives very hard as they keep cutting the amount they will repay GP and kept decreasing the medicine costs and consult costs. Some patients on TPA only pay $2 for consult costs! How can GP survive?! …” (P69, male, private)*

Besides the financial impact, the low fees were also perceived to be disrespectful of GPs’ professionalism.

*“Insurance and Third-party Administrators belittle GPs by paying insultingly low consultation fee to service their clients. It is as little as $6 and on average not more that $10 after taking into account the "admin fee" they charge GPs. This is an insult to GPs and belittling the contributions and professional status of GPs.” (P345, male, private)*

TPA agreements include constraints on fees and clinical procedures, which can compromise the quality and continuity of patient care.

*“Less dealings with TPA insurances which cap the amount of things we can do for patients (patients have to pay out of pocket for uncovered or capped items). A lot of patients (almost 90%) are using TPA insurance and they just hop to whichever clinic is covered by their insurance. So, there is no continuity of care once the insurance company switches their clinics every few years.” (P259, male, public)*

Many felt that TPAs were able to exert their negative influence because they were insufficiently regulated. There were calls for direct government regulation: *“The interference of practice and the exploitation by TPAs must be removed or much more regulated” (*P126, male, private), particularly from those private solo GPs whose caseload was mainly patients from TPAs and who have weak negotiating power. There were numerous calls to remove TPAs altogether: “*Get rid of TPAs. They are basically taking advantage of the lack of governance. It’s not helping either the patients or GP in the long run” (P70, male, private)* and even to “ban” TPAs outright: *“Ban Third Party Payment Schemes. It is a nightmare and the Govt does not want to take a look at it.”* (P10, male, private). There were also calls to make TPAs jointly liable for poor patient outcomes that occur due to the terms in their contract*: “making third party vicariously liable for poor patient outcomes as a result of them limiting or rationing clinical care.”* (P74, male, public).

## Discussion

Doctors in GPFM were moderately satisfied with some aspects of their careers (rapport with patients and current professional role) but less satisfied with other aspects (status of GPFM in society, paperwork and TPA involvement). It is important to be attentive to job satisfaction because higher job satisfaction is linked to a lower turnover intention [4, 22].

The career plans of the GPs surveyed indicate room for boosting retention, though the situation is less dire than that in some countries. In the next 5 years, 14.4% planned to leave GPFM permanently, 12.6% planned to take a career break, and 51.3% planned to reduce clinical hours. Overall, these numbers compare favorably to numbers reported the UK, where only 48.5% intended to work for at least 5 years [23]. These numbers are also slightly more optimistic than the 30% attrition intention among obstetrics and gynaecology residents [24] and the 17% actual attrition among anaesthesia residents [25], both reported in Singapore studies. Perhaps unsurprisingly, the GPs planning to increase their teaching, training, and research responsibilities was higher in the public sector than in the private sector, consistent with the fact that the former work setting offers more opportunities for a varied career portfolio.

The GPs who intended to leave practice permanently in the next 5 years (approximately one in seven) rated time spent on unimportant tasks, reduced job satisfaction, long working hours, and lack of professional support as the most important factors contributing to their plans to leave. In terms of retaining doctors in practice, GPs in both public and private sectors viewed increasing legal protection for medical practitioners and increasing the status of GPFM (through higher remuneration and recognition as a specialty within medicine) as the most important factors. A substantial number of GPs complained about the damaging impact of third-party administrators (TPAs) in hurting practice finances, belittling their profession, and compromising the quality of patient care. Comparing across sectors, doctors in the public sector attached more importance to the need to reduce clinical workload to retaining GPs.

### Comparison with existing literature

The overall picture in our data suggests that Singapore as of now is not experiencing a “GP workforce crisis” or severe GP shortage of the magnitude of several other countries [26, 27]. A recent systematic review that included 25 studies worldwide showed that approximately half of the GPs expressed intentions to leave their current post [4]. A UK survey conducted in 2017 showed that only about 48.5% of GPs surveyed intend to work for at least 5 years [23]. A recent survey from China showed that in 2017/18 over 70% of the GPs reported moderate or strong turnover intentions. While turnover intentions among Singapore GPs are currently lower, our findings show similar sources of dissatisfaction to that reported elsewhere, such as dissatisfaction with salary, low satisfaction with certain aspect of the job, and low level of morale (at least in regard to certain features that are perceived to be not within their control), concerns about legal risks [7]. Our findings on low satisfaction with paperwork burden, long working hours, intensity of workload, and fear of litigation are also consistent with the findings of a UK study on why GPs leave practice early [28]. Given that Singapore government is proposing to upscale the contribution of primary care to achieve a “Healthier SG”, there is an urgent need to be attentive to the current areas of dissatisfaction highlighted in our study when expanding the workforce in order to avoid repetition of the retention challenges faced in other countries.

### Strengths and limitations

The strengths of this study include its use of a variety of question formats including multiple-choice and free-text input questions to generate rich as well as rigorous data, complementing earlier publications focusing on only one-to-one interviews. Participants were sampled from both the public and private sectors in proportion with the general distribution, giving the data representativeness and generalizability for Singapore. It may also be useful to other urban Asian settings as it is one of the few Asian studies of GPs’ career satisfaction and plans outside of China, with Japan being a rare exception [29].

The relatively low response rate is a limitation of this study; despite many efforts to improve this, such as contacting multiple public agencies and networks to distribute the survey and cold-calling GP clinics to invite participation in the study, it was lower than that reported for some other studies (e.g., 54.7% [23] and 67.0% [5] in the UK). However, low response rates among general practitioners not unusual for online surveys of this nature [30] and have been dampened further by the COVID-19 pandemic [31, 32], which considerably reduced the capacity of GPs to participate in research and the capacity of agencies to distribute the survey to GPs. Given that we investigated only career intentions rather than actual behavior, future research, preferably with longitudinal designs, can investigate how much of the leave intentions translates into actual behavior, and how these outcomes are influenced by changes in the practice environment and prevailing sentiments. In addition, we were not able to validate the principal component analysis within the same dataset due to sample size restrictions. The component structure we found awaits validation in future studies.

### Implications for policy and practice

Our findings highlight many issues that need addressing to retain GPs. Across public and private sectors, addressing legal protection was rated as the most important factor for retaining doctors in practice. This sense of vulnerability might have been fueled by the spate of high-profile rulings by the Courts and the Singapore Medical Council’s disciplinary tribunal in the recent years [33]. Suggested solutions include boosting education on medical ethics and laws (preferably taught by plaintiff lawyers to provide a perspective that may avoid lawsuits from the outset) in medical schools [34] and increasing the procedural transparency in the Medical Council’s disciplinary rulings [35]. An excessive fear of malpractice can lead to overtreatment and defensive medicine [36], which drives healthcare costs up and weakens the doctor-patient relationship.

Another point of contention for GPs in both sectors is the status of GPs in the society. Recognition is one of the most important determinants of GP recruitment and retention [37]. The free-text responses suggest that recognition of GPs’ clinical expertise from other specialists and from patients are also important for them to adequately fulfill their roles. Lack of respect from specialist colleagues and lack of confidence from patients is not conducive for integrating the primary, secondary, and tertiary care in a healthcare system facing a growing burden of chronic illnesses.

Not recognizing family medicine as a specialty in its own right has been a longstanding problem in Singapore, despite the Ministry of Health’s recognition that Singapore needs more generalists rather than organ specialists [38]. Recently, there was public denigration of GPs when some doctors including but not entirely GPs expressed concerns about effects of mRNA vaccines on children. The commentary affirmed the position that GPs “were not chosen to be specialists”, “look after everyday illnesses” and refer to specialists illnesses too complex for themselves to manage [39]. This commentary prompted many responses and eventually a letter from the Ministry of Health, College of Family Physicians, and the Singapore Medical Association to clarify that family medicine, which is practiced by GPs, is an established clinical discipline [40]. Unfortunately, specialty training for GPs in Singapore remain nonmandatory unlike in many other countries, reinforcing its perceived inferior status. To overcome this obstacle, mandatory specialty training and recognizing GPFM as a medical specialty can go hand in hand in elevating the status of the field. The profile of family medicine needs to be raised, together with the status of primary care physicians, if Singapore is to derive maximum benefit from primary care [10].

Respondents highlighted their considerable dissatisfaction with third-party administrators (TPAs) and managed care organizations (MCOs) among private GPs and a handful of public GPs. The complaints chiefly centered on how the terms of the TPA agreements have resulted in unacceptably low fees, professional belittlement, and compromises on patient care. Such dissatisfaction has been longstanding, as revealed by the Managed Care surveys conducted by the Singapore Medical Association and the College of Family Physicians Singapore in 2003, 2006 and 2015, in which over half of the doctors surveyed in 2015 were either dissatisfied or very dissatisfied with the MCOs, and 60% thought that their operation in Singapore should cease [41]. Still, many clinics, especially the newly established, enter into agreements with TPAs to boost patient volume. As profit-driven entities, TPAs have been criticized for lacking transparency, prioritizing business interests over patient care quality, having outsized influence on patient treatment while not being regulated as healthcare institutions, and engendering a variety of ethical issues in medical practice [42, 43]. While the Singapore Medical Council has prohibited doctors from entering into TPA arrangements that amount to “fee-splitting” since 2017, other drawbacks of TPA agreements remain [12]. Our findings resonate with this prevailing sentiment and suggest that direct regulatory intervention is required to address this source of dissatisfaction and potential trigger for leaving family medicine. Such regulation may include ensuring a minimum fee for consultation and easing the constraints on clinical procedures that are imposed by TPA agreements. Doing so could also improve evidence-based practice and the quality of patient care.

GPs working in the public sector were particularly dissatisfied with their long working hours, heavy workload, and short consultation times with patients. This is consistent with the reports in the Health Ministry’s primary care survey report [14]. where public sector GPs have an average daily workload of 58 consultations, almost 50% higher than the average daily workload of 39 consultations by their private counterparts, who have more autonomy in managing their workload. Given that many patients still prefer to visit polyclinics because of the subsidized medical fees, limited consultation time afforded in polyclinics may be detrimental for management of complex conditions, which require longer consultations. Therefore, managing polyclinic doctor’s workload to allow for longer consultation times can improve the quality of care delivery. GPs in the private sector, on the other hand, were particularly dissatisfied with career progression opportunities. Guidance on how private GPs can develop “portfolio careers”, which may include education, management, business and research activities, can enrich and advance their careers.

## Conclusion

While primary care in Singapore is not facing an imminent physician shortage, our findings do suggest that much needs to be done to improve the engagement of GPs, facilitate expansion, and improve the quality of care. With one in four people in Singapore expected to be aged 65 or above by 2030, caring for a population with multiple complex chronic illnesses and with higher expectations will be an unprecedented challenge for primary care doctors. Effectively realizing the vision of “Three Beyonds” by the Ministry of Health [44] require a committed and motivated primary care physician workforce. The key areas for action are stronger legal protection, increasing regulation of TPAs and MCOs, and improving the status and recognition of GPFM. Enabling and supporting this workforce in navigating these challenges is essential for meeting the healthcare needs of an aging population.

## Data Availability

The datasets and survey questions can be accessed at https://doi.org/10.21979/N9/62LHEC.
